# The Drosophila Gene *Sulfateless* Modulates Autism-Like Behaviors

**DOI:** 10.3389/fgene.2019.00574

**Published:** 2019-06-19

**Authors:** Kevin A. Hope, Daniel Flatten, Peter Cavitch, Ben May, James S. Sutcliffe, Janis O’Donnell, Lawrence T. Reiter

**Affiliations:** ^1^Integrated Program in Biological Sciences, The University of Tennessee Health Science Center, Memphis, TN, United States; ^2^Department of Neurology, The University of Tennessee Health Science Center, Memphis, TN, United States; ^3^Christian Brothers University, Memphis, TN, United States; ^4^Rhodes College, Memphis, TN, United States; ^5^Department of Molecular Physiology and Biophysics, Vanderbilt University School of Medicine, Nashville, TN, United States; ^6^Department of Psychiatry, Vanderbilt University School of Medicine, Nashville, TN, United States; ^7^Department of Biological Sciences, University of Alabama, Tuscaloosa, AL, United States; ^8^Department of Anatomy and Neurobiology, The University of Tennessee Health Science Center, Memphis, TN, United States; ^9^Department of Pediatrics, The University of Tennessee Health Science Center, Memphis, TN, United States

**Keywords:** DGRP, autism, Drosophila, heparin sulfate, GWAS

## Abstract

Major challenges to identifying genes that contribute to autism spectrum disorder (ASD) risk include the availability of large ASD cohorts, the contribution of many genes overall, and small effect sizes attributable to common gene variants. An alternative approach is to use a model organism to detect alleles that impact ASD-relevant behaviors and ask whether homologous human genes infer ASD risk. Here we utilized the Drosophila genetic reference panel (DGRP) as a tool to probe for perturbation in naturally occurring behaviors in *Drosophila melanogaster* that are analogous to three behavior domains: impaired social communication, social reciprocity and repetitive behaviors or restricted interests. Using 40 of the available DGRP lines, we identified single nucleotide polymorphisms (SNPs) in or near genes controlling these behavior domains, including ASD gene orthologs (*neurexin 4* and *neuroligin 2*), an intellectual disability (ID) gene homolog (*kirre*), and a gene encoding a heparan sulfate (HS) modifying enzyme called *sulfateless* (*sfl*). SNPs in *sfl* were associated with all three ASD-like behaviors. Using RNAi knock-down of neuronal *sfl* expression, we observed significant changes in expressive and receptive communication during mating, decreased grooming behavior, and increased social spacing. These results suggest a role for HS proteoglycan synthesis and/or modification in normal social communication, repetitive behavior, and social interaction in flies. Finally, using the DGRP to directly identify genetic effects relevant to a neuropsychiatric disorder further demonstrates the utility of the Drosophila system in the discovery of genes relevant to human disease.

## Introduction

Major hurdles to understanding the pathophysiology of autism spectrum disorder (ASD) include extreme genetic heterogeneity combined with small effect sizes for most risk genes, and the inherent difficulty in identifying non-genetic factors (e.g., environmental and epigenetic) that may increase autism risk. Approximately 20–30% of cases of non-syndromic ASD correspond to individually rare, highly penetrant, often *de novo*, variants. These variants range from “pathogenic” to significant risk variants of large effect and can show incomplete penetrance, pleiotropy or both (De Rubeis et al., 2014; Sanders et al., 2015). *Rare* genic variants include both copy number variants (CNVs), a loss or gain of DNA ≥ 1000 bp and more discrete, single nucleotide variants (SNVs), or small indels detected from whole exome or whole genome sequencing. Genome wide association studies (GWAS) use genotype arrays to capture common genetic variation across the genome and test for association with disease using family-based or case-control designs. This approach has been remarkably successful in schizophrenia for which combined samples in the psychiatric genomics consortium (PGC) have surpassed 50,000 and have identified well over 150 genome-wide significant loci (Li et al., 2017; Pardinas et al., 2018). By comparison, the most recent ASD GWAS from the PGC identified only a few genome-wide significant loci (Autism Spectrum Disorders Working Group of the Psychiatric Genomics Consortium, 2017), but amalgamated ASD cohorts are not yet of the size to reach the critical “inflection point” previously observed for schizophrenia (Schizophrenia Working Group of the Psychiatric Genomics Consortium, 2014).

In contrast, a combination of whole exome and genome sequencing and CNV discovery in family and case-control samples has identified a number of genes disrupted by rare, damaging, *de novo* variants, and highlighted biology and function that resonates across psychiatric disorders, like RNAs regulated by Fragile X mental retardation 1 (FMRP) (Iossifov et al., 2012), synaptic scaffolding proteins, chromatin regulating machinery, and other genes related to overlapping neuropsychiatric disorders. A major hindrance to the success of GWAS in identifying associations to ASD and other complex behavioral disorders is the small effect sizes conferred by common variants (Devlin et al., 2011). Mouse models of single gene or contiguous gene models of autism have been useful in some cases, for example, Rett syndrome (*Mecp2*) (Liao et al., 2012) and duplications of human 15q11.2-q13 (Nakatani et al., 2009; Smith et al., 2011). In general, however, these models have a limited utility for understanding the multigenic nature of ASD, since they model only single, highly penetrant mutations. Here we propose a new approach to analyze common variants contributing to autism-like behaviors at multiple loci that conspire to confer ASD risk in humans by using the powerful genetic tools available in the model organism *Drosophila melanogaster*.

In this study, we identified a set of behaviors in flies that may be analogous to the human behaviors associated with ASD. Specifically, we assay (1) social communication during mating (or courtship behavior); (2) repetitive behavior during grooming; and (3) social reciprocity, in a social spacing test. We used these analogous behaviors to query the Drosophila genetic reference panel (DGRP), a community resource consisting of 205 sequenced inbred lines that have been derived from a natural population (Mackay et al., 2012), in order to identify shared common alleles across behaviors. The DGRP lines have already been used successfully to identify new candidate genes that influence a number of Drosophila behaviors and adaptations to environmental stressors (Mackay and Huang, 2018). Here we assessed both individual behavior domains and combined behaviors in order to identify genes that could influence multiple ASD-like fly behaviors. Ultimately, we seek to use this approach to tease apart the complex genetics that lead to autism phenotypes in humans.

## Results

### Selection of Behavior Assays Analogous to ASD Behaviors

We sought to identify measurable fly behaviors that map to the three core ASD phenotypic domains: communication, social interaction and repetitive behaviors, and restricted interests. The current Diagnostic and Statistical Manual for Mental Disorders, 5th edition (DSM-V; American Psychiatric Association, 2013) criteria combines social interaction with communication, creating a category of *social communication*. Although many naturally occurring behaviors can be measured in the fly model, we felt that there were only a few behaviors which resembled human behaviors closely enough to use in this study. The most obvious behavior is a recently published assay for fly social spacing (analogous to human social reciprocity). Humans with ASD often interact with other individuals awkwardly. This includes inappropriate responses to social cues which can cause them to either violate another persons’ “personal space” or to over-react when an individual touches them or invades their personal space. When flies are housed as a group they also settle into a “comfortable” social spacing that can be quantified using the social space triangle (Simon et al., 2012).

Another clearly analogous behavior to human ASD-like behaviors is any measure of repetitive behavior. Repetitive grooming has been used in flies previously as a measure of ASD symptomology in Fragile X mutants (Drozd et al., 2018). Here we use the flies normal grooming behavior as a read out for restrictive and repetitive interests, one of the most common ASD symptoms in humans.

Perhaps the hardest ASD feature to approximate in any animal model are defects in communication, a uniquely human trait that does not translate well to animals that lack a specific language of their own. However, researchers have made attempts to decipher the simple languages of many animals in the wild. Perhaps most well-known animal vocalizations are the various bird calls that can indicate alarm or mating calls or other specific behavioral cues, which may be learned and processed in ways similar to human language [reviewed in Mol et al. (2017)]. Flies also use a primitive version of communication during the mating process (Kamikouchi, 2013; Aranha and Vasconcelos, 2018). Male flies will vibrate their wings to generate a “song” that the female flies must respond to during the mating process. If the male fly does not produce the song correctly, or at all, there will be no mating. Similarly, if the female fly does not respond to the song or interpret the song correctly as a mating call, she may not mate with the male (Greenspan and Ferveur, 2000). Therefore, if we assay the DGRP line in a male during courtship, we are measuring a form of expressive communication (the song) and if we assay a DGRP line in a female to a wild type male during courtship we are measuring a form of receptive communication. Here we use the latency to mating during courtship behavior to quantify these differences in expressive and receptive communication, both prominent features of ASD.

To detect differences in response to *social reciprocity*, we used a relatively new test for social spacing developed in flies. This assay exploits the natural tendency of flies to (1) be negatively geotaxic (i.e., flies tend to move upward) and (2) settle into an average spacing of ∼1.5 fly lengths apart (Simon et al., 2012). Flies were tested in groups of 25, and the average distance between flies in a group was quantified for each DGRP genotype.

To assay *repetitive behaviors/restricted interests*, we utilized a simple grooming behavior test. Flies typically groom for a few seconds when placed into a new environment, so individual DGRP flies being tested were moved into a chamber for video recording of total time spent grooming during a 5 min trial. Each of these tests produced sensitive measures that capture trait variation in DGRP lines analyzed. We conducted a GWAS with the resulting data to identify genes that potentially influence these behaviors, as in previous DGRP studies (Mackay and Huang, 2018).

### GWAS and Analysis of 40 DGRP Lines Revealed Significant Associations

**FIGURE 1:**
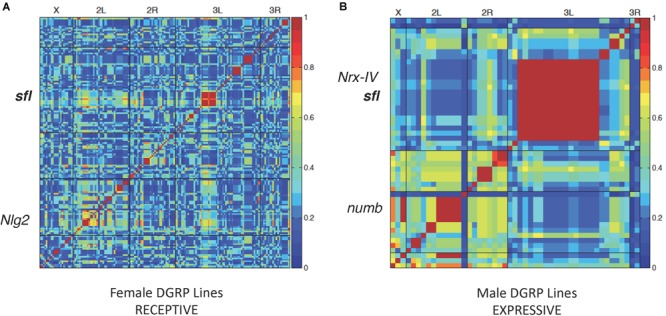
Linkage disequilibrium heatmaps and social communication. LD map of variants associated with female **(A)** or male **(B)** DGRP lines in the mating latency test. Each DGRP line was tested three times and the average mating latency used for LD mapping for 40 lines using the online GWAS tool^2^. Red regions represent significant association between those variants and mating latency. Several variants were in or around known autism associated genes *Nlg2, Nrx-IV*, and *numb*. variants in *sfl* were also associated with mating latency from both the receptive **(A)** or expressive **(B)** side of the mating test.

Forty DGRP lines were picked at random from the collection for testing in all three behavior paradigms. Each line was tested in triplicate for a given behavior, and the results averaged for input into the genome wide association (GWA) calculation. Males and females were scored separately but input together in order to identify male- or female-specific trait effects. A summary of the SNPs and genes identified for each behavior, with mixed *p* value < 0.001, is provided in Table 1. Complete input data for each behavior in all 40 lines and a plot illustrating variation within the population for each behavior, separately in males, and females is provided in Supplementary Data 1. A heatmap of GWAS results for receptive and expressive communication, as measured by mating latency, is presented in Figure 1. We detected 159 variants (SNPs, indels or insertions) in or near several genes that showed significant association with receptive communication (females receptive to the male’s song) including *Neuroligin 2* (*Nlg2*), homologous to the autism associated gene *NLGN3*, and *sulfateless* (*sfl*), a gene that encodes an enzyme involved in heparan sulfate residue synthesis (Figure 1A). Similarly, expressive communication showed strong association with 49 variants, including several in loci on chromosome 3L, such as *neurexin 4* (*Nrx-IV*), orthologous to the ASD involved gene contactin-associated protein 2 (*CNTNAP2*) and again, *sfl*. Also associated was *numb*, homologous to *NUMBL* (*numb*-like), a human gene involved in nervous system development but not implicated in ASD, called *NUMBL* in humans (Figure 1B).

**Table 1:** Summary of GWAS results.

Behavior	SNPs (mixed *p* value < 0.001)	Genes associated
Mating (receptive)	159	83
Mating (expressive)	46	16
Grooming	302	134
Social spacing	51	31

**FIGURE 2:**
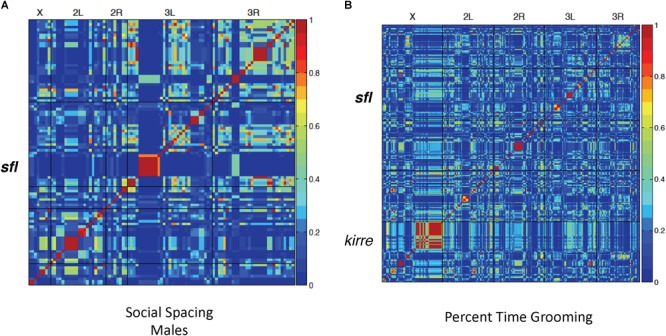
Linkage disequilibrium heatmaps, social spacing and grooming. LD mapping for 40 DGRP lines in both the social interaction (spacing) and grooming tests. Red areas indicate significantly associated variants. **(A)** Using the social spacing triangle, the average distance among flies was measured. The average value for three runs was used for LD mapping for 40 lines using the online GWAS tool (http://dgrp2.gnets.ncsu.edu/). **(B)** Total time grooming was measured for each of 40 DGRP lines in three separate runs and the average value used for LD mapping for 40 lines using the online GWAS tool (http://dgrp2.gnets.ncsu.edu/). In both social spacing tests and grooming tests there was a significant association between SNPs in or around *sfl* and the behavior being measured.

Using social spacing as a proxy for social reciprocity, we identified significant association at 51 variants across the genome, including a single variant located within intron 1 of the *sfl* gene (Figure 2A). Finally, 302 variants were significantly associated with repetitive grooming behavior in the 40 DGRP lines tested (Figure 2B), including the gene *kin of irre* (*kirre*). The homologous human gene *KIRREL3* has been associated with ID (Kalsner et al., 2018). Once again, a SNP included in intron I of the *sfl* gene was associated with repetitive grooming behavior.

**FIGURE 3:**
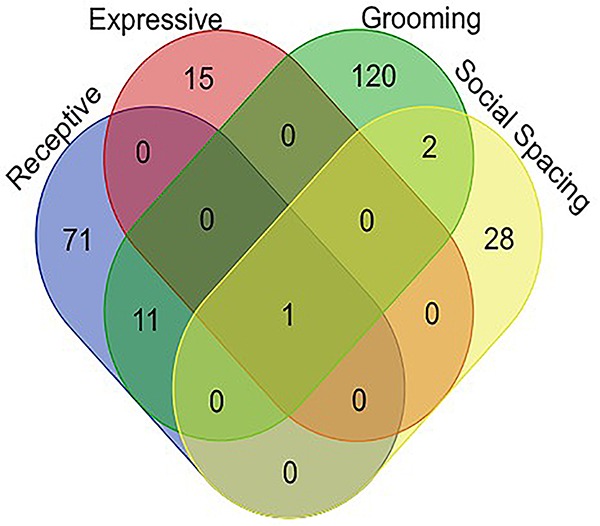
Venn diagram representing all of the significant gene associations for all behaviors measured in 40 DGRP lines. Note that there is only one gene, *sfl*, that showed significant associations for all four ASD-like behaviors. Raw output for all GWAS studies is available in the Supplementary Data Section.

Venn diagram analysis^1^ for associated genes of all four behaviors tested confirmed that the only gene which appears to be associated with social communication, social spacing, and grooming is the sulfotransferase encoding gene, *sfl* (Figure 3). All variants associated with each behavior domain, the minor allele frequencies and the *p*-values for each variant as well as the gene names and potential regulatory effects are listed in the Supplementary Data 2.

Next, we combined measures from all behavioral domains into one ASD-like measure for each DGRP line. We calculated *Z*-scores for each DGRP line across all four behaviors tested and summed these values, producing a single value for each DGRP line. This value represents how far from the mean each DGRP line fell when considering all four behavioral measures combined, a similar approach to how ASD is diagnosed in humans as ASD testing relies on results from multiple behavioral domains. We conducted a GWAS on the combined *Z*-score value for each DGRP line and identified 131 significantly associated variants, including a single variant in located upstream of the *sfl* gene (Supplementary Data 3).

### Knockdown of *sfl* in Neurons Significantly Affects All ASD-Like Behaviors Tested

Since all of the individual behaviors tested, as well as the combined *Z*-score analysis, showed at least one association to *sfl*, we focused functional analysis on experiments designed to test involvement of *sfl* in ASD-relevant behaviors. First, we looked at each variant for a potentially direct role in the regulation of *sfl*. Several transcription factor binding sites for critical developmental factors (*delta, hairless, twist, zeste, giant, trithorax*-like, *hunchback, medea*, and *daughterless*) are located in the upstream region containing SNPs associated with expressive communication. However, none of these transcription factors have been shown to regulate mating behavior previously. There were no other transcription factor binding sites disrupted by *sfl* SNPs identified in this study, and the majority of variants occurred in intragenic or intronic regions and did not obviously disrupt protein coding or gene regulation. All of the SNPs for *sfl* were mapped back to the UCSC genome browser for Flybase Release August 06, 2014 and are illustrated in Supplementary Figure 1.

**FIGURE 4:**
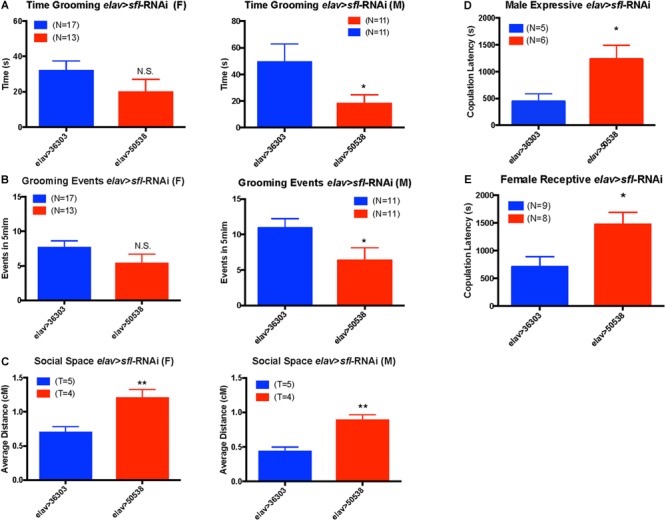
Neuronal knockdown of *sfl* alters ASD-related behaviors. Using a UAS-*sfl*-RNAi line (BDSC #50538) from the TRiP collection (https://fgr.hms.harvard.edu/fly-in-vivo-rnai) we knocked down expression of *sfl* in neurons using pan-neuronal driver *elav*-GAL4. We then assayed each of the four ASD-like behaviors showing significant association with *sfl*. **(A)** Knockdown of *sfl* (elav > *sfl*-RNAi) caused a significant reduction in grooming behavior in males as compared to control crosses to the control TRiP insertion line (*elav* > 36303), but not in females (two-tailed *t*-test *p* value ≤ 0.05). **(B)** There was also a significant reduction in the number of grooming events in males where *sfl* was knocked down (*elav* > *sfl*-RNAi), but not females (two-tailed *t*-test *p* value ≤ 0.05). **(C)** Social spacing was significantly increased for both males and females in *elav* > *sfl*-RNAi flies (*elav* > 50538) as compared to the RNAi control line (*elav* > 36303) (two-tailed *t*-test, *p* value ≤ 0.05). T, number of trails using 25 flies each trial. **(D)** Knock down of *sfl* in males mated to Canton-S females (expressive communication) caused a significant increase in latency to mating (two-tailed *t*-test, *p* ≤ 0.05). **(E)** Knock down of *sfl* in females mated to Canton-S males (receptive communication) also caused a significant increase in mating latency (*t*-test, *p* ≤ 0.05). ^∗^*p* ≤ 0.05, ^∗∗^*p* ≤ 0.01, and N.S., not significant.

Given the association between all behaviors tested and *sfl*, we used the GAL4/UAS system (Duffy, 2002) to knock down *sfl* expression in neurons using UAS-RNAi and the pan-neuronal driver, *elav*-GAL4. We then looked for significant changes in any of these behaviors vs. control [empty attP recombination site for the TRiP project (Hu et al., 2017)]. For each behavior we measured males and females separately to account for sex specific effects. We found a significant decrease in the time spent grooming in *elav* > *sfl*-RNAi males, but not females (two-tailed *t*-test *p* value ≤ 0.05) (Figure 4A). Similarly, we saw a decrease in the number of grooming events in males, but not females with knockdown for *sfl* in neurons (two-tailed *t*-test *p* value ≤ 0.05) (Figure 4B). We also saw a significant increase in social spacing for both males and females in *elav* > *sfl*-RNAi flies (two-tailed *t*-test *p* value ≤ 0.05) (Figure 4C). Finally, we found that both expressive and receptive communication during mating behavior is disrupted by knock down of *slf* in neurons (Figure 4D,E). Male *elav > sfl*-RNAi flies had significantly longer copulation latencies compared to *elav > 36303* control males when paired with *Canton-S* wildtype virgin female flies (*t*-test, *p* = 0.0317). Similarly, female *elav > sfl-*RNAi flies displayed significantly longer copulation latencies compared to *elav > 36303* flies when paired with virgin male *Canton-S* flies (*t*-test, *p* = 0.0157). These results show a clear causation between decreased *sfl* expression in neurons and all three ASD-related behavior domains tested.

## Discussion

### Drosophila as a Model System for Human ASD

Simple behaviors in *D. melanogaster* have been used to model human conditions for over a century (Ugur et al., 2016). Early studies of epilepsy, neurodegeneration and circadian rhythm may be the most familiar examples. However, after we discovered a strong molecular connection between human disease associated genes and the fly genome [namely that 75% of the genes known to cause human disorders have homologs in flies (Reiter et al., 2001)] there has been renewed interest in utilizing this easily tractable genetic model organism specifically for the study of human disorders, even among researchers unfamiliar with the abundant genetic tools available in flies (Wangler et al., 2015). In terms of this study, an interest in Fragile X syndrome modeling in flies by many labs somewhat parallels our use of behaviors in flies to approximate ASD-like behaviors (Drozd et al., 2018). In particular, one group utilized grooming behavior, as we did, as a read out for ASD-like repetitive behavior (Tauber et al., 2011). In fact, the fly social spacing assay we use here (Simon et al., 2012) has been used to assess a curious phenomenon in humans with ASD, specifically that the age of the father increases the risk of ASD in offspring (Brenman-Suttner et al., 2018). What is unique about our study is that we have combined these previously published ASD assays as a screening tool for the DGRP in order to evaluate the influence of multiple ASD related genes at once, which may be difficult in other systems, either mice or human GWAS, because they are not single genes with strong effects, but rather have more subtle genetic influence on the individual ASD-like behaviors and the combined *Z*-score across these behaviors.

Here we used an un-biased GWA approach in Drosophila to detect genes involved in regulating behaviors relevant to ASD. The DGRP lines used in this study allow for the analysis of naturally occurring behaviors in animals that represent the genetic variation of individuals from a wild caught population with little to no background effects and complete genome sequences for all 205 DGRP lines (Mackay et al., 2012). Using only 40 of the 205 DGRP lines available, we identified associations between three ASD-related behaviors: social communication during mating, social reciprocity during the social space test, and repetitive behavior during grooming. Importantly, our analysis implicated several known ASD genes in regulating these behaviors, providing validity to the approach. Moreover, this analysis revealed a single novel gene, *sfl*, that influences all three behavioral domains.

While there are now many single genes, especially many new *de novo* mutations, known to affect ASD behavior in humans, we were not looking for a single gene at the onset of this study. Our hypothesis was that multiple genes would have subtle effects on ASD behavior and would be difficult, if not impossible, to identify in mice or humans using GWAS, but could be potentially be identified using the power of the DGRP system vs. the more complex genomes of mice or humans. We were, in fact, surprised to find a single gene affecting all three domains tested since we expected a more cumulative effect of multiple genes across all of the domains. The *Z*-score approach, which also revealed the influence of *sfl* across all the behaviors tested, was the original intention of the project. However, we soon realized that finding individual genes affecting specific ASD-like domains like grooming behavior or social communication were also interesting results. Although the finding of a single gene that can influence all three behavioral domains was unexpected, the mechanism by which a human heparan sulfate (HS) N-sulfotransferase may be involved in ASD phenotypes in humans is not. HS proteoglycans are involved in many aspects of neural development including neurogenesis, axon guidance, and synaptogenesis (Yamaguchi, 2001). Furthermore, loss of HS has been firmly linked to ASD-like phenotypes in murine models. The mouse knock-out for *exostosin-1* (*Ext1*), a gene encoding an HS-modifying enzyme essential for the production of HS in the brain, also shows strong ASD-like behaviors including impaired social interaction and increased repetitive behaviors as well as impaired ultrasonic vocalization (Yamaguchi, 2001). Furthermore, a SNP in *EXT1* was the most significant genic association (2.94 × 10^-7^) in a recent GWAS from the ASD working group of the PGC (Autism Spectrum Disorders Working Group of the Psychiatric Genomics Consortium, 2017).

In addition to *sfl*, we identified an association between combined *Z*-score and the non-synonymous coding variant M330L in the gene *Fps85D*, also known as *FER tyrosine kinase*. DGRP lines carrying the lysine variant tended to have higher *Z*-scores (Supplementary Data 3), and thus increases in the behavioral measures, compared to those carrying the methionine variant. These data suggest that the expression or function of *Fps85D* also may impact the behavioral domains we tested. Additional studies will be required to confirm this hypothesis.

The canonical BTBR mouse model (*BTBR T +tf/J*) has been used extensively to study ASD behaviors. BTBR mice have been shown to lack fractal-like structures (fractones) in the sub-ventricular zone of the brain (Meyza et al., 2012). These fractones carry HS cargo to various locations in the brain, thus depriving the developing brain of a key component required for proper neural development and synaptogenesis (Irie et al., 2012). Finally, studies on postmortem human ASD brain samples using immunofluorescence to detect changes in HS in the sub-ventricular zone recapitulate the findings from the BTBR mouse model, showing decreased levels of HS in ASD brain vs. controls (Pearson et al., 2013). While there is no direct evidence to suggest that the human ortholog of *sfl, NDST2*, is itself an ASD gene, the highly conserved HS functions between flies and humans point, in this case, to a shared process associated with ASD, if not a specific gene. Our study now connects these changes in HS levels in neurons directly to the behaviors of social communication, repetitive behavior, and social reciprocity through an HS modifying enzyme encoded by *sfl*.

The implications here are that even subtle changes in HS modifying enzymes or HS proteoglycan synthesis may have a large effect on behavior. One possible reason for this is the diversity of proteoglycan chains that can be created in order to specify appropriate interactions between cells in the brain. In fact, mutations in the cell membrane bound glypican 4 (GCP4) protein have been found in ASD (Doan et al., 2016) and it would not be surprising to find other HS proteoglycans associated with human psychiatric conditions based on their roles in synaptogenesis (Condomitti and de Wit, 2018).

The DGRP collection was primarily designed as a tool to investigate naturally occurring variation in a single population. Our study is the first to utilize the DGRP for the direct study of behaviors related to autism, but not the first to use this powerful tool for the study of human genetic disease. This collection has been used to successfully identify modifiers of neurodegeneration in a fly Parkinson’s disease model (Lavoy et al., 2018) and also candidate modifiers of retinitis pigmentosa using an F1 crossing strategy in combination with the GAL4/UAS system (Chow et al., 2016).

### Caveats and Future Directions

In this study, we used a GWA approach in conjunction with the DGRP to identify candidate genes for regulating ASD-related behaviors in the fly. One major caveat to the study is that we selected naturally occurring behaviors in flies that may or may not be analogous to ASD behaviors in humans, although the detection of associations with know ASD associated genes lends confidence in the use of these behavioral traits. We also are aware that there are several ways in which the individual behaviors tested here could yield false positive or false negative results. For example, we used mating behavior as a proxy for social communication and assume that the defects in latency to mating are due to defects in male song or female reception to the mating song. Another possibility, however, is that the flies being tested for a given DGRP line could have locomotor defects that cause them to take longer to mate, or cause them to not mate at all. It is unlikely that these animals do not copulate, however, since each DGRP line can be maintained as an isogenic stock. Similarly, in the grooming behavior, flies with severe motor defects may want to groom excessively, but simply cannot do so due to physical limitations, although we are confident that we would have easily observed such global motor defects had they been present. In the end, such movement defects could eliminate a few individual DGRP lines from the analysis, but it should not alter the positive results we found for associations between a given behavior and particular genes, especially those where multiple SNPs were found in or near a given gene.

Although RNAi knock down experiments in neurons confirmed a role for *sfl* in all three behaviors, it is likely that a larger GWA screen using all 205 sequenced lines would reveal additional genes associated with all three behavior domains (and even more affecting two behavioral domains only). Our secondary analysis of all three behavior domains at once using normalized *Z*-scores may be a more useful direct link to ASD than the genes found in the individual association studies. While *sfl* was the only gene found in both the three behavioral domains and the combined *Z*-score analysis, which is why we focused on *sfl* for secondary validation studies, other genes were identified in the *Z*-score analysis that may similarly, contribute to relevant behaviors that require further testing. Future studies should include more detailed analysis of HS residues in postmortem human brain as well as additional candidate gene approaches focused on HS synthesis and modification genes to identify risk variants in human ASD cohorts. Finally, additional validation studies using RNAi knock-down or over-expression in neurons to verify the involvement of many of the candidate genes from this study could be performed to expand the list of ASD associated candidate genes identified using this approach.

## Materials and Methods

### Fly Stocks

All stocks used in this study came from the Bloomington Stock Center (BDSC) including the Genetic Reference Panel (DGRP) screening kit, the control Canton S line (64349), TRiP UAS-RNAi lines for *sfl* (50538), and the control TRiP insertion site (36303). The pan neuronal *elav*-GAL4 stock came from Dr. Hugo Bellen, Baylor College of Medicine, Houston, TX, United States. All flies were grown on standard corn meal agar media (BDSC) and kept at 25°C in a humidified environmental chamber on a 12 h day/night cycle.

### Behavioral Testing

Mating, grooming, and social spacing behaviors have all been previously described (Greenspan and Ferveur, 2000; Simon et al., 2012). For mating and grooming assays, single flies or pairs of flies (one male and one female) were video recorded using an Apple iPhone 5 for later analysis. Average latency to mating (seconds) was scored as the time until the male fly successfully mounted the female fly and was recorded for each line for both expressive and receptive social communication. Total average time grooming in a 5 min interval was used for repetitive behavior. For social spacing, 25 flies were used in each experiment (all of the same genotype and sex) and the flies were allowed to equilibrate in the social space triangle for 1 h prior to photographing with an Apple iPhone 5. ImageJ was used to calculate the nearest neighbor distance for each individual fly and nearest neighbor distances were averaged within each experiment.

A total of 40 DGRP lines were selected at random from the collection of 205 lines and used for all three behavior tests. Each individual test on each DGRP line was performed three independent times to obtain and average performance value on that test (latency to mating, time grooming, or social spacing). Supplementary Data 1 contains all of the average input values for each DGRP line used in this study. These average values were input into the GWAS tool (males and females listed separately). The output of the GWAS tool^2^ provides a large amount of data including the heatmaps illustrated in Figure 1, 2 as well as the table output data (Allele frequencies, sex effects, location of associated SNPs and other information) available in Supplementary Data 2 (each Excel Tab represents GWAS for a single behavior domain). A complete set of all raw data used to calculate the average values for input into GWAS (individual animals tested or individual trails for social spacing) is compiled in Supplementary Data 4.

### *Z*-Score Analysis

A summed *Z*-score was calculated for each DGRP line to combine measures from all behavioral domains into one integrated score. *Z*-scores were calculated by finding the mean and standard deviation for each behavioral assay (separating male and females for grooming and social space), and then calculating how many standard deviations from the mean each DGRP line fell. *Z*-scores were summed across all behavioral assays to give each DGRP line a summed *Z*-score. The summed *Z*-score was used for GWAS analysis^2^.

### *sfl* Knockdown Experiments

Using the GAL4/UAS system (Duffy, 2002) and a UAS-RNAi line (BDSC #50538) available from the Harvard TRiP collection, expression levels of *sfl* were knocked down in all neurons using the *elav*-GAL4 driver. The control line, BDSC# 36303, contains an attP40 insertion site without the UAS-RNAi insert and is used as a control for experiments utilizing these TRiP lines. Female *elav*-GAL4 virgins were crossed to males for the TRiP collection and the offspring allowed to mature at least 3 days prior to behavior testing. All animals were reared in an incubator on a 12 h day/night cycle. Behavior tests were performed as described above using at least 10 animals per genotype for grooming and mating behaviors and at least 4 independent runs (25 flies each run) for the social spacing behaviors. Statistical analysis was performed using Prism 7.0 to calculate the standard error of the mean, significance by Student’s *t*-test (*p* value ≤ 0.05) and graph the results.

## Author Contributions

KH designed and executed the experiments, interpreted the data, and prepared the manuscript. DF, PC, and BM executed the experiments and interpreted the data. LR and JO’D developed the concept, managed the project, interpreted the data, and prepared the manuscript. JS interpreted the data and prepared the manuscript.

## Conflict of Interest Statement

The authors declare that the research was conducted in the absence of any commercial or financial relationships that could be construed as a potential conflict of interest.

## Footnotes

1 http://bioinformatics.psb.ugent.be/webtools/Venn/

2 http://dgrp2.gnets.ncsu.edu/
