# Supplementary material for: The Drosophila Gene Sulfateless Modulates Autism-Like Behaviors
Source: Front Genet. 2019 Jun 19;10:574. doi: 10.3389/fgene.2019.00574 (PMC6611434; doi:10.3389/fgene.2019.00574)
Supplement: Supplementary file 2 [file Data_Sheet_2.PDF]

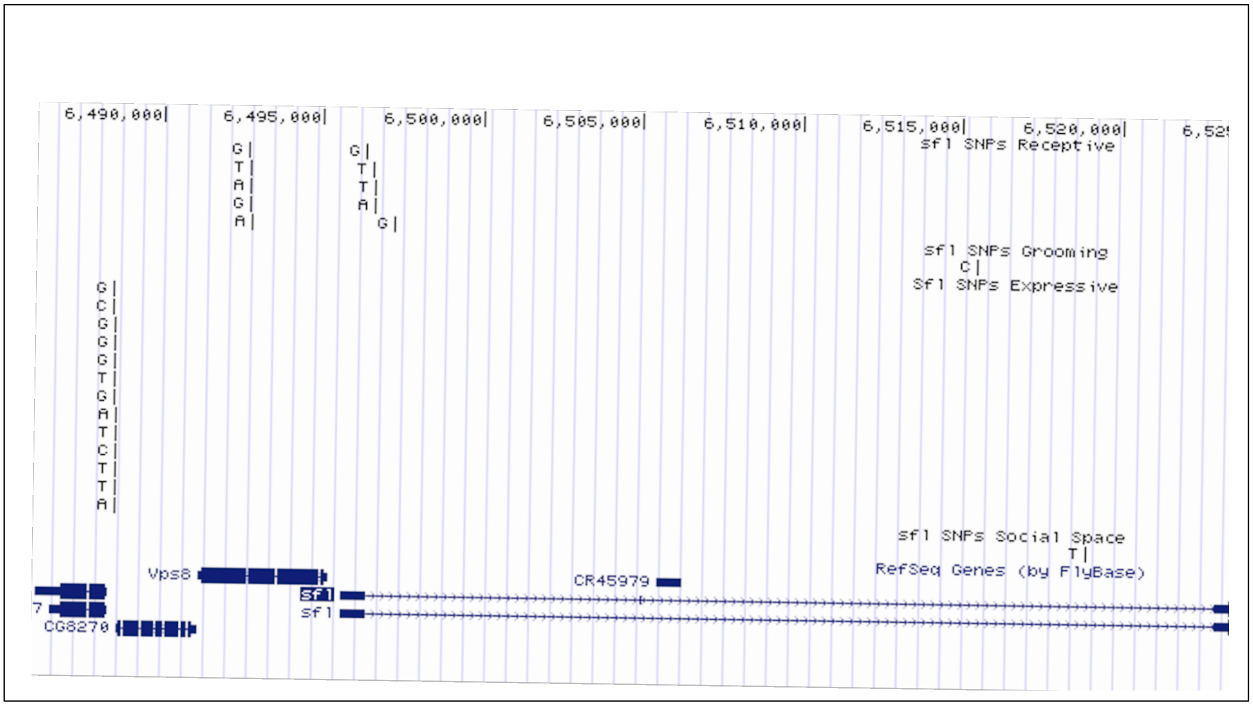

Supplemental Fig 1. Location of SNPs in or around *sfl* linked to ASD-like behaviors. A) Using a BED file with the locations of all SNPs associated with *sfl* for each behavior a genomic region diagram was constructed within the UCSC genome browser for *D. melanogaster* (Aug. 2014). B) Higher magnification of this region confirms that none of the SNPs are within coding regions of *sfl* and some SNPs are located in the intergenic space between two genes upstream of *sfl*.

Supplemental Data Files (.zip file) contains:

Supplemental Data 1. Excel sheets with averaged input values for GWA in Mating Latency, Grooming and Social Spacing.

Supplemental Data 2. Excel sheets with output from the GWA tool for significantly associated SNPs in each behavior, gene lists used to construct the Venn, SNPs located in or around *sfl* and BED file of *sfl* SNPs.

Supplemental Data 3. Excel sheets with output from the GWA tool on the Z-score analysis for significantly associated SNPs.

Supplemental Data 4. Excel sheets with all raw data collected for Mating Latency, Grooming and Social Spacing.
